# Supplementary figures and images for: Targeting corticotropin-releasing hormone receptor type 1 (Crhr1) neurons: validating the specificity of a novel transgenic Crhr1-FlpO mouse
Source: Brain Struct Funct. 2024 Dec 18;230(1):12. doi: 10.1007/s00429-024-02879-0 (PMC11655595; doi:10.1007/s00429-024-02879-0)

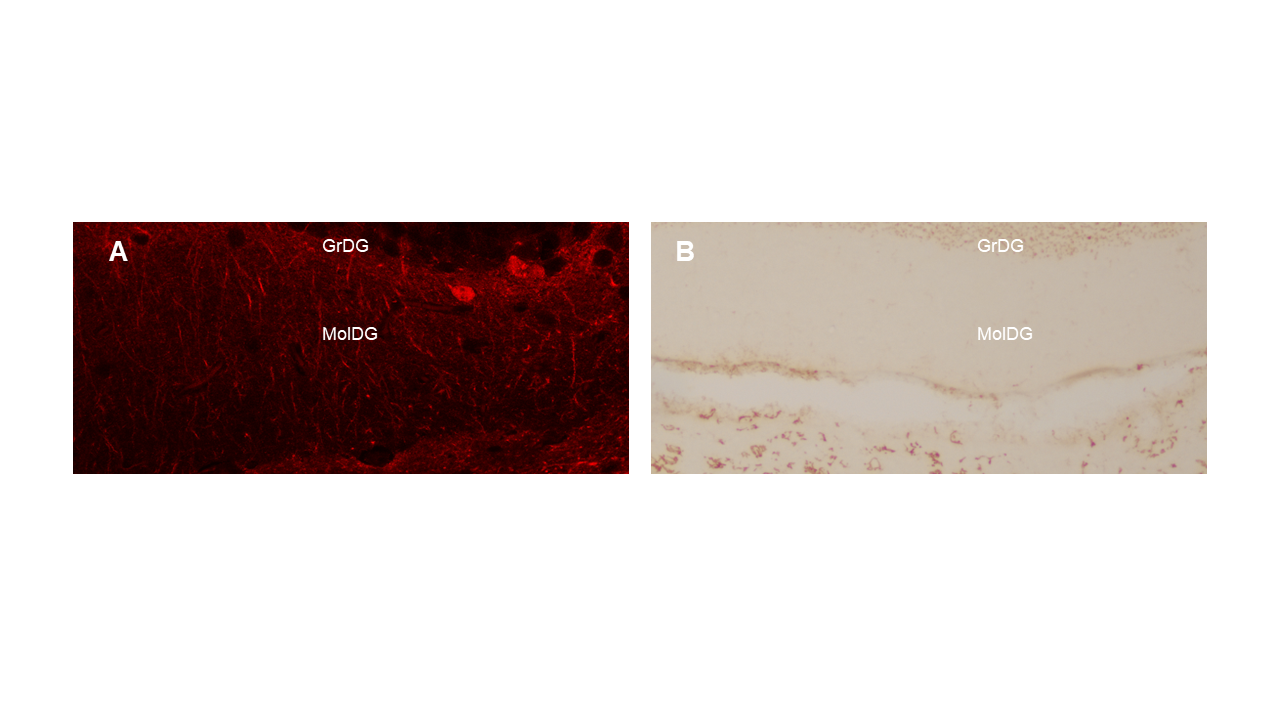

Supplement: Supplementary file 1 — Supplementary Material 1 [file 429_2024_2879_MOESM1_ESM.tif]

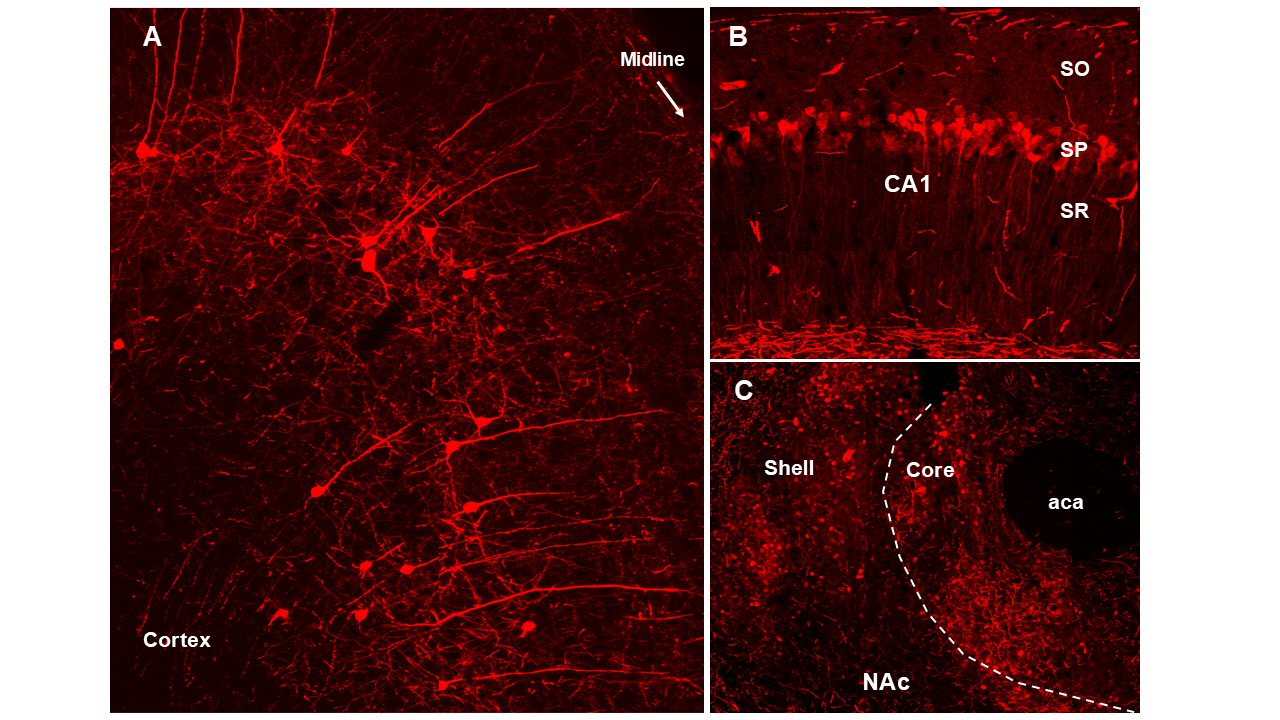

Supplement: Supplementary file 2 — Supplementary Material 2 [file 429_2024_2879_MOESM2_ESM.tif]
